# Development of 17 novel microsatellite markers for *Lycoris aurea* and *L. radiata* (Amaryllidaceae) using next‐generation sequencing

**DOI:** 10.1002/aps3.1198

**Published:** 2018-11-14

**Authors:** I‐Ju Chen, Chou‐Tou Shii, Tsu‐Liang Chang, Kae‐Kang Hwu

**Affiliations:** ^1^ Department of Horticulture and Landscape Architecture National Taiwan University No. 1, Sec. 4, Roosevelt Road Taipei 10617 Taiwan; ^2^ Department of Agronomy National Taiwan University No. 1, Sec. 4, Roosevelt Road Taipei 10617 Taiwan

**Keywords:** Amaryllidaceae, genetic variation, hybrid, *Lycoris aurea*, *Lycoris radiata*, microsatellite

## Abstract

**Premise of the Study:**

*Lycoris* is an ornamental and medicinal plant. We developed microsatellite markers for *L. aurea* and *L. radiata* simultaneously by using a hybrid between these two species.

**Methods and Results:**

Ion Torrent next‐generation sequencing produced 1,784,504 reads. Testing 64 primer sets allowed for the development of 17 novel microsatellite markers: 16 for *L. aurea*, 10 for *L. radiata*, and nine common markers. *Lycoris aurea* had one to 12 alleles per locus and observed and expected heterozygosity levels of 0–0.923 and 0.038–0.809, respectively. *Lycoris radiata* had three to 12 alleles per locus and observed and expected heterozygosity levels of 0–0.909 and 0.127–0.797, respectively. Ten markers were cross‐amplified for *L. sprengeri*.

**Conclusions:**

Hybrid sequencing can facilitate the cost‐effective development of molecular markers for parental species. The markers developed here are useful for studying *Lycoris* population structure.

The genus *Lycoris* Herb. comprises more than 20 species and is endemic to eastern and southern Asia. *S*pecies within *Lycoris* have potential commercial value due to their unusual flower shape and summer to autumn flowering habit, when few other bulbous flowers are available in the market (Hsu et al., 1994). Some alkaloids produced by *Lycoris* (e.g., galantamine) have been used clinically to treat symptoms of Alzheimer's disease, and the alkaloid lycorine has anticancer and antiviral activity (Takos and Rook, 2013).

The first simple sequence repeat (SSR) markers for *Lycoris* (16 polymorphic markers) were generated using *L. longituba* Y. Hsu & G. J. Fan expressed sequence tags (ESTs) (He et al., 2009). Ten more markers were obtained from *L. radiata* (L'Hér.) Herb. using an enriched genomic library, some of which were successfully amplified in *L. sprengeri* Comes ex Baker, *L. anhuiensis* Y. Hsu & G. J. Fan, *L. albiflora* Koidz., *L. longituba*, and *L. chinensis* Traub (Xuan et al., 2011). However, for the *L. radiata* samples tested in the present study, the 12 markers developed by He et al. (2009) and the markers developed by Xuan et al. (2011) produced null or more than two alleles, or heavy stutter that likely interfered with the distinction of adjacent alleles (Appendices S1 and S2). Many SSRs have been found in the 454 sequenced EST cDNA library of *L. aurea* (L'Hér.) Herb. (Wang et al., 2013); however, there have been no reports regarding the success of the cross‐amplification of these repeats in *L. radiata*.

*Lycoris aurea*,* L. radiata*, and *L. sprengeri* are native to Taiwan, whereas *L. radiata* and *L. sprengeri* are highly restricted in Lianjian County, which is composed of a group of islets in proximity to mainland China. However, their natural population size has been greatly reduced due to habitat changes. Here, we report a set of SSR markers developed from a hybrid between *L. aurea* and *L. radiata*; these markers were polymorphic for both parental species and showed cross‐amplification with *L. sprengeri*. These SSR markers will be useful for population studies and the conservation of these species.

## METHODS AND RESULTS

A hybrid obtained by crossing *L. aurea* and *L. radiata* was shotgun‐sequenced using an Ion Torrent (Personal Genome Machine) platform (Thermo Fisher Scientific, Waltham, Massachusetts, USA) to produce 1,784,504 reads. The 1,326,609 reads longer than 50 bp (average length 247.3 bp) were screened for SSRs using Tandem Repeat Finder 4.07b (Benson, 1999). Although dinucleotide repeats were the most abundant type of repeats, they were excluded because they frequently have severe stutter bands with high repeat numbers (Guichoux et al., 2011). Because the predicted SSR variability is positively related to the repeat number and purity (Legendre et al., 2007), only 570 reads that displayed a repeat purity >90% and repeat numbers ≥7 (tri‐ and tetranucleotides) and ≥8 (pentanucleotides) were further analyzed. To reduce size variation (Meglécz et al., 2010), flanking sequences containing microsatellites and homopolymers with more than five repetitions were trimmed using an in‐house ad hoc R script, resulting in 293 reads, for which 208 primer pairs were designed using BatchPrimer3 (You et al., 2008). Primer sequences were checked against raw reads using BLAST+ (Camacho et al., 2009), and primers occurring more than once were discarded because they likely originated from duplicated genomic regions or organelles. Finally, 64 of the remaining 98 microsatellites with more than eight repeat units were validated by PCR amplification and capillary electrophoresis.

Fresh leaf samples were freeze‐dried or silica gel–dried. Genomic DNA was extracted using a modified cetyltrimethylammonium bromide (CTAB) protocol (Doyle and Doyle, 1990) and then stored at −20°C. The multiplex‐ready system (Hayden et al., 2008) was used for testing polymorphisms, and PCR amplifications were performed using the following four primers in a single reaction: specific forward and reverse primers tagged at their 5′ ends with 5′‐ACGACGTTGTAAAA‐3′ and 5′‐GTTTAAGTTCCCATTA‐3′, respectively; forward universal tag primer, 5′‐ACGACGTTGTAAAA‐3′, labeled with PET, VIC, NED, or 6‐FAM fluorescent dye at its 5′ terminus (Applied Biosystems, Waltham, Massachusetts, USA); and a reverse universal tag primer, 5′‐GTTTAAGTTCCCATTA‐3′, with a PIG‐tailing modification (Brownstein et al., 1996). The total reaction volume of 10 μL contained 20 ng of DNA template, 1× ImmoBuffer (Bioline, London, United Kingdom), 1.5 mM magnesium chloride (MgCl_2_), 0.2 mM dNTPs, 0.25 units *Taq* DNA polymerase (Bioline), 40 nM forward‐specific primer, 40 nM reverse‐specific primer, 80 nM labeled fluorescence tag forward primer, and 80 nM universal tag reverse primer. Amplifications were conducted on a thermal cycler (GeneAmp PCR System 9700, Applied Biosystems) using the following conditions: 95°C for 10 min; 20 cycles at 92°C for 30 s, 63°C for 90 s, and 72°C for 60 s; 40 cycles at 92°C for 15 s, 54°C for 30 s, and 72°C for 60 s; and 72°C for 30 min. Capillary electrophoresis was performed using a Genetic Analyzer 3730 (Applied Biosystems), and SSR fragment analysis was performed using GeneMapper version 4.0 (Applied Biosystems).

All samples were collected from Taiwan. The *L. aurea* samples included two collections from natural populations found at BiTou Cape in New Taipei City and Taroko in Hualien County, as well as a long‐term collection cultivated in the National Taiwan University greenhouse (NTU collection). The *L. radiata* samples included three collections from natural populations found at Mo Tian Ling, Tie Ban, and Da Niu Shan in Nangan Township, Lianjiang County, and a private collection made by the Coast of the Dawn nursery (COD collection) from the islets of Lianjiang County. *Lycoris sprengeri* from Dongyin Township, Lianjiang County, was tested for cross‐amplification. Geographical and voucher information are provided in Appendix 1.

The 64 SSR primer pairs were screened with eight *L. aurea* and eight *L. radiata* samples. The 29 and 17 primer pairs amplifiable for *L. aurea* and *L. radiata*, respectively, were subsequently tested with extended sample sets (48 from each species), resulting in 17 SSR markers that were clearly scored and produced no null alleles for one or both species (Table 1). Among the 16 and 10 markers that were amplifiable for *L. aurea* and *L. radiata*, respectively, nine were amplifiable for both species. Cross‐amplification tests run for the 17 SSR markers using 10 *L. sprengeri* samples revealed that 10 markers were cross‐amplifiable for *L. sprengeri* and eight markers were cross‐amplifiable for all three species.

**Table 1:** Characteristics of the 17 microsatellite loci developed from the hybrid of *Lycoris aurea* and *L. radiata* and variation in *L. aurea*,* L. radiata*, and *L. sprengeri*

Locus	Primer sequences (5′–3′)	Repeat motif	*L. aurea* (*N* = 70)		*L. radiata* (*N* = 95)		*L. sprengeri* (*N* = 10)		GenBank accession no.
			*A*	Allele size (bp)^a^	*A*	Allele size (bp)^a^	*A*	Allele size (bp)^a^	
LAR_001	F: GCCATGGAAGCTGGAGTAGA	(GAA)_11_	8	146–170	12	141–173	3	139–151	Pr032825052
	R: AAAGGAAGCCTAACTTCAAATTCA								
LAR_037	F: GGCATATGGGGGAATTGTTA	(TCT)_10_	3	132–144	10	135–166	4	130–142	Pr032825055
	R: GCATAAAATTACAGTGCAGATGCT								
LAR_038	F: TAAGCCCCTTTGCCCTAAAT	(TCC)_10_	12	209–239	8	209–236	5	215–233	Pr032825056
	R: ACACCACCTCCCAGAACTTG								
LAR_060	F: GGACGAAACTAAGAATGCATGTG	(TCT)_9_	8	194–214	8	192–210	2	204–207	Pr032825059
	R: TGCTAGACAAAGCAGCGACT								
LAR_084	F: ATATGCGAGAAGCCTGGAGA	(ATG)_9_	7	124–142	4	112–121	2	121–124	Pr032825060
	R: AGCGTTGCTTTGTAGCCAAT								
LAR_152	F: GCGGCGGAGAGTAGTAAGTG	(AGG)_8_	9	252–271	8	255–279	3	249–262	Pr032825064
	R: TCTTATCAATCGCCACGTCA								
LAR_164	F: GCGGTTGAAATCTTTACAAATCA	(TCA)_8_	1	195	5	186–198	2	186–189	Pr032825066
	R: TAGAGGCAAAGGAGCCCATA								
LAR_194	F: GGTGCAACTTTTTCCTTCCA	(GAA)_9_	4	156–165	5	146–152	1	143	Pr032825068
	R: CCATTGACCAAGGACAAACC								
LAR_029	F: CATGCAATATTCCAAACAAGGA	(GAA)_10_	2	138–143	3	137–143		—	Pr032825054
	R: TTGATCGAATACTTTCTTCAAATG								
LAR_054	F: GGGTTTTCCTGTTTGCACTC	(AGA)_10_		—	6	173–197	2	161–164	Pr032825058
	R: AAATGTGAGAACCGGTCTGG								
LAR_155	F: GGGAGACGATAGCAATGACG	(ATT)_9_	1	199		—	3	192–198	Pr032825065
	R: TCCCATACTTCCAAAACCAAA								
LAR_003	F: TGACCCTTACAGGTTCCATTTT	(CTT)_12_	9	101–128		—		—	Pr032825053
	R: GAATGAGTAAATGCAGGAGAGGA								
LAR_053	F: TTGTTGGGCTCTTCCATAGG	(TCA)_9_	3	161–167		—		—	Pr032825057
	R: GCTCGATGGAGTTGGGAATA								
LAR_091	F: TCCACGATTTTGTCACTCTGA	(ATT)_11_	7	182–206		—		—	Pr032825061
	R: TGCGCTTTTCTTTCTTTTTACC								
LAR_107	F: GGGTAACGCAAGTGCTTGAT	(GAA)_8_	3	164–168		—		—	Pr032825062
	R: AAGCCCCATCTTCTTTTGGT								
LAR_141	F: TGTATGAGGAAGGAATCAAGGAA	(TAT)_9_	7	170–188		—		—	Pr032825063
	R: TCCTTGACCACTGGTGGATT								
LAR_179	F: CCTTTTAGCCACGTCAAACC	(TAAA)_8_	7	129–141		—		—	Pr032825067
	R: CATGGCCAGTGAATTTGAGA								
Mean			5.7		6.9		2.7		

Note

*A* = number of alleles; *N* = sample size.

a Sizes include forward and reverse universal primers; — signifies no product or a missing rate >10%.

Individuals from two natural populations of *L. aurea* and three natural populations of *L. radiata* exhibited similar genetic complexity, with an average number of alleles per locus ranging from 3.8 to 4.1 (Tables 2 and 3). The COD collection of *L. radiata* had an exceedingly high average number of alleles per locus (5.3), indicating that this collection comprised plants collected over a wide range of natural populations. The number of alleles per locus, observed and expected heterozygosity, and Hardy–Weinberg equilibrium were estimated using GenAlEx 6.5 (Peakall and Smouse, 2012). With a few exceptions, the observed heterozygosity was smaller than the expected heterozygosity when differences were statistically significant (Tables 2 and 3), which indicated that population size reductions might have caused population inbreeding.

**Table 2:** Genetic diversity of 16 microsatellite loci in three populations of *Lycoris aurea*.^a^

Locus	BiTou Cape (*n* = 26)			Taroko (*n* = 21)			NTU collection (*n* = 23)		
	*A*	*H*_o_ b	*H*_e_	*A*	*H*_o_ b	*H*_e_	*A*	*H*_o_ b	*H*_e_
LAR_001	5	0.615^ns^	0.632	7	0.381^*^	0.533	6	0.435^**^	0.594
LAR_037	2	0.038^ns^	0.038	3	0.143^ns^	0.135	1	—	—
LAR_038	6	0.923^ns^	0.801	10	0.857^*^	0.781	9	0.826^***^	0.809
LAR_060	6	0.154^***^	0.749	5	0.381^ns^	0.661	5	0.087^***^	0.374
LAR_084	5	0.500^***^	0.699	6	0.381^ns^	0.548	4	0.348^*^	0.589
LAR_152	4	0.385^ns^	0.595	6	0.286^***^	0.497	5	0.348^***^	0.701
LAR_164	1	—	—	1	—	—	1	—	—
LAR_194	4	0.615^ns^	0.652	3	0.286^ns^	0.500	4	0.304^***^	0.615
LAR_029	1	—	—	1	—	—	2	0.043^ns^	0.043
LAR_155	1	—	—	1	—	—	1	—	—
LAR_003	6	0.500^ns^	0.624	6	0.571^***^	0.772	5	0.478^*^	0.558
LAR_053	3	0.154^ns^	0.144	2	0.238^ns^	0.210	3	0.043^***^	0.198
LAR_091	6	0.692^ns^	0.743	6	0.571^**^	0.760	4	0.739^ns^	0.610
LAR_107	2	0.115^***^	0.440	2	0^***^	0.172	3	0.087^***^	0.436
LAR_141	4	0.038^***^	0.212	4	0.190^**^	0.464	3	0.043^***^	0.084
LAR_179	4	0.308^***^	0.331	3	0.524^ns^	0.516	6	0.652^**^	0.751
Mean	3.8	0.315	0.416	4.1	0.301	0.409	3.9	0.277	0.398

Note

*A* = number of alleles; *H*_e_ = expected heterozygosity; *H*_o_ = observed heterozygosity; *n* = number of individuals.

a Voucher and locality information are provided in Appendix 1.

b Significant deviation from Hardy–Weinberg equilibrium: *P < 0.05, **P < 0.01, and ***P < 0.005; ns = not significant.

**Table 3:** Genetic diversity of 10 microsatellite loci in four populations of *Lycoris radiata*.^a^

Locus	Mo Tian Ling (*n* = 22)			Tie Ban (*n* = 26)			Da Niu Shan (*n* = 21)			COD collection (*n* = 26)		
	*A*	*H*_o_ b	*H*_e_	*A*	*H*_o_ b	*H*_e_	*A*	*H*_o_ b	*H*_e_	*A*	*H*_o_ b	*H*_e_
LAR_001	5	0.273^***^	0.691	6	0.731^**^	0.797	7	0.667^ns^	0.664	8	0.538^***^	0.776
LAR_037	6	0.909^**^	0.685	6	0.192^**^	0.338	6	0.333^***^	0.687	7	0.269^***^	0.476
LAR_038	4	0.500^ns^	0.640	4	0.769^ns^	0.663	6	0.714^ns^	0.675	7	0.615^ns^	0.741
LAR_060	6	0.227^***^	0.749	4	0.192^ns^	0.371	5	0.143^***^	0.405	6	0.346^ns^	0.496
LAR_084	3	0.591^ns^	0.439	3	0.462^ns^	0.462	3	0.476^ns^	0.550	4	0.231^***^	0.575
LAR_152	5	0.591^ns^	0.655	6	0.692^ns^	0.691	3	0.476^ns^	0.520	4	0.385^*^	0.510
LAR_164	2	0.136^ns^	0.127	1	—	—	1	—	—	5	0.154^ns^	0.146
LAR_194	3	0.182^ns^	0.305	3	0.154^***^	0.322	2	0.095^***^	0.363	5	0.269^***^	0.574
LAR_029	3	0^***^	0.376	3	0.115^***^	0.354	3	0^***^	0.322	3	0.038^***^	0.177
LAR_054	4	0.545^ns^	0.427	4	0.692^*^	0.730	4	0.524^ns^	0.670	4	0.462^ns^	0.607
Mean	4.1	0.395	0.509	4.0	0.400	0.473	4.0	0.343	0.486	5.3	0.331	0.508

Note

*A* = number of alleles; *H*_e_ = expected heterozygosity; *H*_o_ = observed heterozygosity; *n* = number of individuals.

a Voucher and locality information are provided in Appendix 1.

b Significant deviation from Hardy–Weinberg equilibrium: *P < 0.05, **P < 0.01, and ***P < 0.005; ns = not significant.

## CONCLUSIONS

We developed 17 novel microsatellite markers for *Lycoris* and demonstrated that the analysis of short sequence reads obtained from the hybrid used here might be a useful way to discover SSR markers for both parental species. By excluding flanking sequences containing microsatellites and homopolymers, and by choosing motifs with three or more nucleotides, we achieved a high amplification rate, while avoiding the stutters that are commonly associated with dinucleotide repeats. The markers developed in this study are useful additions for studying the population structure of *L. aurea* and *L. radiata* and, to some extent, *L. sprengeri*.

## DATA ACCESSIBILITY

Raw sequencing reads were deposited in the National Center for Biotechnology Information (NCBI) Sequence Read Archive (accession no. PRJNA493520). Sequence information for the developed primers has been deposited to NCBI's GenBank; accession numbers are provided in Table 1.

## Supporting information


**APPENDIX S1.** Success or failure rate and failure reasons for the 10 markers developed in *Lycoris* by Xuan et al. (2011), using 13 *Lycoris radiata* samples.

**APPENDIX S2.** Electropherogram profiles of fluorescence‐labeled SSR products of the 10 markers developed in *Lycoris* by Xuan et al. (2011), showing overlap in 13 samples (column A) and one sample that failed severely (column B).

## APPENDIX 1 Voucher and locality information for *Lycoris* species used in this study.

**Table:**

Species	Collection locality^a^	*n*	Geographic coordinates	Voucher collection no.	Herbarium ID^b^
*Lycoris aurea* (L'Hér.) Herb.	BiTou Cape, New Taipei City	26	25°07′22.9″N, 121°54′59.8″E	L0436	TAI 286824
	Taroko, Hualien County	21	24°10′23.7″N, 121°32′43.2″E	L0517	TAI 286830
	National Taiwan University, Taipei City	23	25°00′54.9″N, 121°32′21.1″E	L0471	TAI 286847
*Lycoris radiata* (L'Hér.) Herb.	Mo Tian Ling, Nangan Township, Lianjiang County	22	26°09′58.1″N, 119°57′24.2″E	L0268	TAI 286825
	Tie Ban, Nangan Township, Lianjiang County	26	26°08′25.4″N, 119°55′27.5″E	—	—
	Da Niu Shan, Nangan Township, Lianjiang County	21	26°09′19.6″N, 119°57′13.9″E	—	—
	Coast of the Dawn nursery, Nangan Township, Lianjiang County	26	26°08′36.7″N, 119°55′39.1″E	L0167	TAI 286846
*Lycoris sprengeri* Comes ex Baker	Suicide Cliff, Dongyin Township, Lianjiang County	10	26°22′09.6″N, 120°30′27.9″E	L0193	TAI 286826

Notes

*n* = sample size.

a All samples were collected from Taiwan.

b One voucher was collected from each sampled population except in the Tie Ban and Da Niu Shan area, where only leaf collection was permitted. The vouchers were deposited in the Herbarium (TAI), Institute of Ecology and Evolutionary Biology, National Taiwan University, Taipei, Taiwan.
