# Supplementary material for: Development of 17 novel microsatellite markers for Lycoris aurea and L. radiata (Amaryllidaceae) using next‐generation sequencing
Source: Appl Plant Sci. 2018 Nov 14;6(11):e01198. doi: 10.1002/aps3.1198 (PMC6240451; doi:10.1002/aps3.1198)
Supplement: Supplementary file 1 — APPENDIX S1. Success or failure rate and failure reasons for the 10 markers developed in Lycoris by Xuan et al. (2011), using 13 Lycoris radiata samples. [file APS3-6-e01198-s001.docx]

APPENDIX S1. Success or failure rate and failure reasons for the 10 markers developed in *Lycoris* by Xuan et al. (2011), using 13 *Lycoris radiata* samples.

| Marker | Success rate (%) | Failure rate  (%) | Failure reason | | |
| --- | --- | --- | --- | --- | --- |
|  |  |  | Multi-locus | Stutter | Null |
| Lyra-1 | 69.23 | 30.77 | 0.00 | 15.38 | 15.38 |
| Lyra-2 | 0.00 | 100.00 | 0.00 | 100.00 | 0.00 |
| Lyra-3 | 7.69 | 92.31 | 0.00 | 76.92 | 15.38 |
| Lyra-4 | 0.00 | 100.00 | 0.00 | 100.00 | 0.00 |
| Lyra-5 | 7.69 | 92.31 | 7.69 | 84.62 | 0.00 |
| Lyra-6 | 0.00 | 100.00 | 0.00 | 61.54 | 38.46 |
| Lyra-7 | 15.38 | 84.62 | 0.00 | 84.62 | 0.00 |
| Lyra-8 | 15.38 | 84.62 | 0.00 | 84.62 | 0.00 |
| Lyra-9 | 7.69 | 92.31 | 0.00 | 84.62 | 7.69 |
| Lyra-10 | 0.00 | 100.00 | 100.00 | 0.00 | 0.00 |
