# Supplementary material for: Development of 17 novel microsatellite markers for Lycoris aurea and L. radiata (Amaryllidaceae) using next‐generation sequencing
Source: Appl Plant Sci. 2018 Nov 14;6(11):e01198. doi: 10.1002/aps3.1198 (PMC6240451; doi:10.1002/aps3.1198)

Chen et al.—Applications in Plant Sciences 2018 6(11)—Data Supplement S2.
DOI

Appendix S2. Electropherogram profiles of fluorescence-labeled SSR products of the 10 markers developed in *Lycoris* by Xuan et al. (2011), showing overlap in 13 samples (column A) and one sample that failed severely (column B).

A **B**

Lyra-1

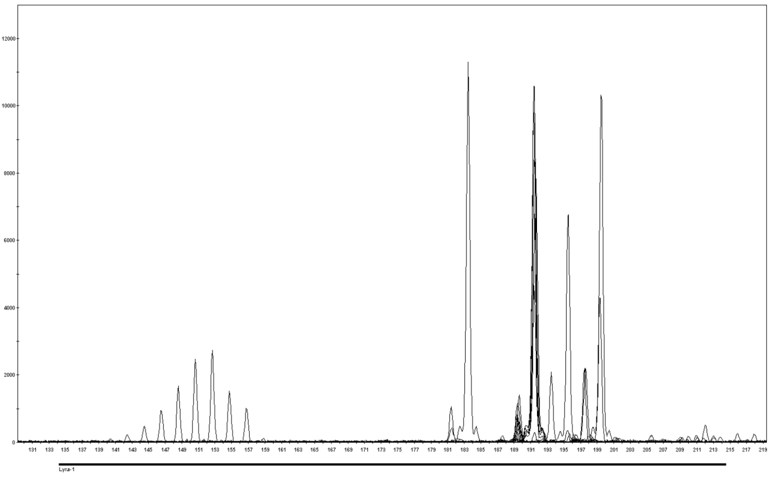

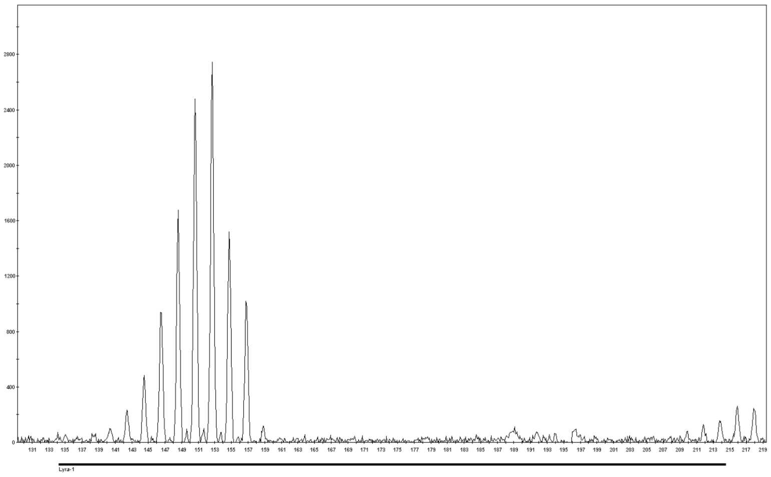

(DNA ID: 317)

Lyra-2

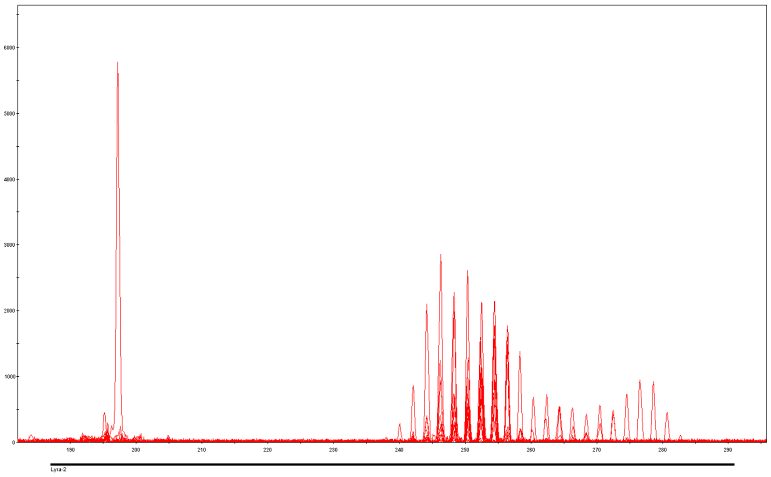

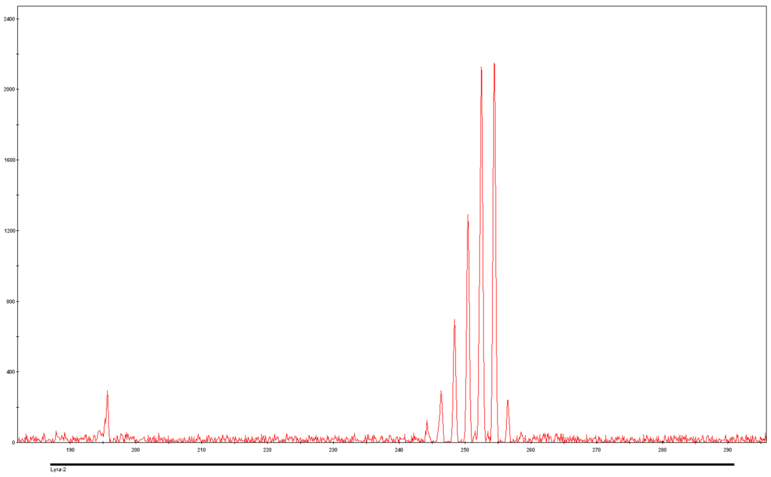

(DNA ID: 317)

Chen et al.—Applications in Plant Sciences 2018 6(11)—Data Supplement S2.
DOI

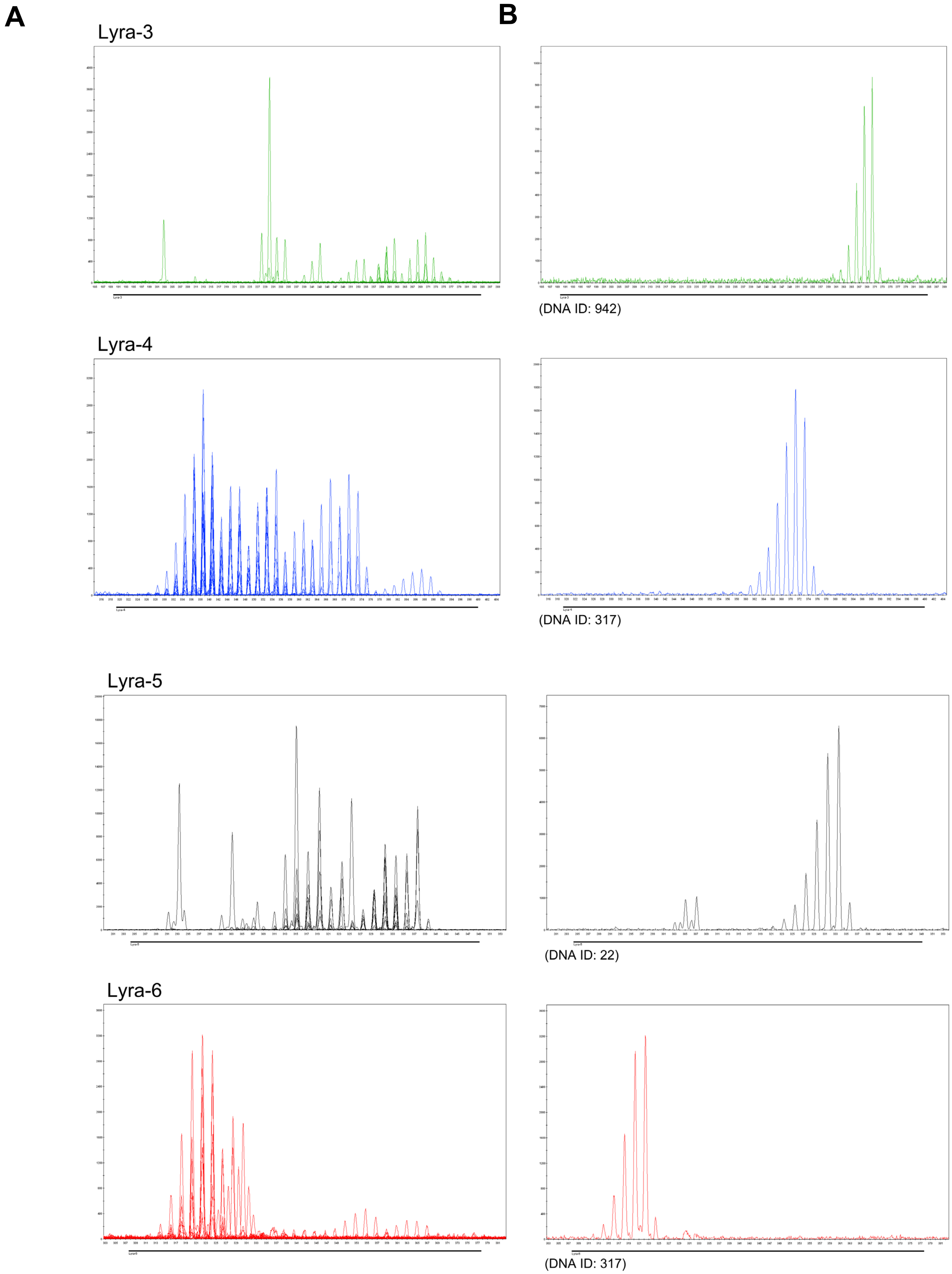

Chen et al.—Applications in Plant Sciences 2018 6(11)—Data Supplement S2.
DOI

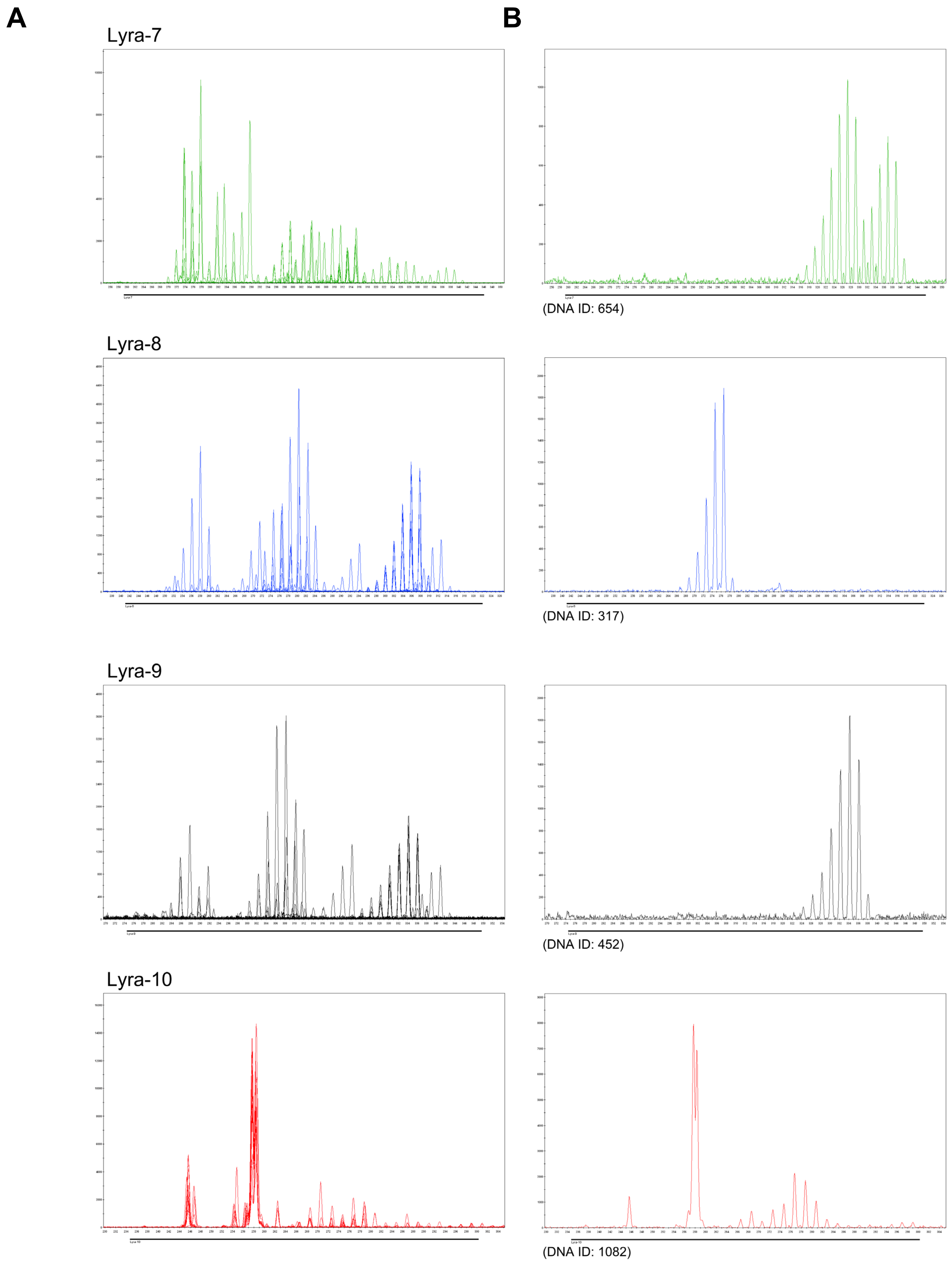

Supplement: Supplementary file 2 — APPENDIX S2. Electropherogram profiles of fluorescence‐labeled SSR products of the 10 markers developed in Lycoris by Xuan et al. (2011), showing overlap in 13 samples (column A) and one sample that failed severely (column B). [file APS3-6-e01198-s002.pdf]
